# Thermal imaging for sealing defect detection in pharmaceutical bags using a temporal fusion network

**DOI:** 10.1371/journal.pone.0343395

**Published:** 2026-03-09

**Authors:** Liqiang Wang, Ziyang Leng, Cunmin Jiang, Rui Hua

**Affiliations:** 1 Rongchang Pharmaceuticals (Zibo) LTO, Zibo, China; 2 School of Artificial Intelligence, Anhui University, Hefei, China; Newcastle University, UNITED KINGDOM OF GREAT BRITAIN AND NORTHERN IRELAND

## Abstract

Sealing defects in pharmaceutical plastic bags pose significant risks to drug safety, as micro-leakages may remain undetected until transportation, causing economic losses and hazards. Traditional manual inspection and existing automated methods suffer from low efficiency, poor sensitivity to subtle defects, and difficulties in addressing class imbalance due to scarce defective samples. To address these issues, this study proposes a comprehensive detection framework that integrates thermal imaging analysis, physics-guided data augmentation, and a novel Temporal Multi-Feature Fusion Network (TMFFNet). Thermal imaging reveals defective areas with distinct localized temperature elevations, providing a reliable basis for defect identification. A physics-guided augmentation method is developed to synthesize realistic defects: it models defect contours via hybrid polynomials, simulates thermal diffusion using dual-Gaussian operators, and fuses synthetic defects into normal samples under geometric constraints. This method effectively mitigates class imbalance, expanding the number of defective samples from 28 real ones to 2104 synthetic ones, with a total of 4385 samples in the dataset. The proposed TMFFNet, a dual-branch temporal network, processes three consecutive thermal frames to capture temporal dynamics. Its global-local fusion module enhances sensitivity to small defects, while a channel-aware SE-Dense module suppresses background noise, reducing false alarms. Experimental results show that TMFFNet outperforms traditional networks with a test set accuracy of 0.9809, and other evaluation metrics also demonstrate favorable performance. This framework provides an efficient, non-destructive solution for full pharmaceutical packaging inspection, improving drug safety and production efficiency.

## Introduction

In the pharmaceutical production process, the sealing quality of drug packaging has always been a key link in ensuring product safety and effectiveness [1–3]. In particular, for plastic-sealed bags containing liquid drugs, tiny defects in the heat-sealed areas may lead to slow leakage. Such problems often only become apparent during transportation rather than immediately after production, and the resulting economic losses and safety risks cannot be ignored.

Currently, the inspection of packaging quality for liquid preparations mainly relies on manual squeezing sampling: operators manually squeeze the sampled products and judge whether there is liquid leakage through visual observation. This method has extremely low inspection efficiency and cannot realize full inspection. Moreover, long-term repetitive operations are prone to fatigue, with strong subjectivity, leading to high rates of missed detections and misjudgments. In addition, the plastic-sealed bags are small in size and the leakage rate is slow; some subtle sealing defects are difficult to manifest through short-term squeezing. This results in serious deficiencies in inspection reliability and traceability. With the improvement of automation level in pharmaceutical production lines, manual sampling inspection can no longer meet the needs of modern pharmaceutical industry for efficient, non-destructive, and real-time inspection. Among existing inspection technologies, optical cameras are difficult to identify micro-gap leakage, while X-ray equipment has the problems of high cost, large volume, and potential impact on the stability of preparations. Furthermore, the number of defective samples for sealing inspection of liquid pharmaceutical preparations is scarce, resulting in class imbalance. Generation-based methods can generate new samples by learning data distribution, such as DCGAN network [4] and FastGAN network [5]. Dai et al. [6] proposed a framework to improve the performance of spot welding defect classification via GAN-based data augmentation, based on the BAGAN-GP network. This framework utilizes BAGAN-GP to generate diverse minority-class images. Zhang et al. [7] proposed a lightweight defect classification method based on few-shot image generation and self-attention fused convolutional features. To tackle the problem of limited defect samples, this method first expands defect images through geometric enhancement techniques and then further augments the dataset using GANs. Such a dataset augmentation approach has achieved favorable results. However, such methods are time-consuming in training, require high computing resources, and are difficult in parameter adjustment, leading to poor applicability in industrial inspection. Oversampling techniques such as SMOTE [8–10] can synthesize minority class samples through interpolation at a low cost, but they tend to generate blurred samples.

In recent years, the development of convolutional neural networks (CNNs) has advanced by leaps and bounds. The evolution from traditional convolutional neural networks to lightweight ones has made it possible to deploy models for classification tasks under resource-constrained conditions [11–13]. Moreover, classification networks represented by convolutional neural networks (CNNs) have been increasingly widely applied in defect detection [14–16]. Kabir A. Pathak et al. [17] based on Convolutional Neural Networks, manually created defective samples for the defect detection of film-coated tablets in pharmaceutical production. Ajantha Vijayakumar et al. [18] proposed the CBS-YOLOv8 model, incorporating BiFPN to enhance feature fusion across convolutional layers and improving the model’s efficiency by implementing SimSPPF. Liu et al. [19] conducted semantic segmentation work on surface defects of aluminum-plastic blister packaged pharmaceuticals based on convolutional neural networks, which effectively enhanced the capability of multi-scale defect detection and the accuracy of defect boundaries. However, with the introduction of Vision transformer [20], an increasing number of people have been using the attention mechanism in their work. Yi et al. [21] conducted the work of locating and classifying foreign particles in liquid pharmaceuticals, based on the multi-scale attention-based feature fusion (MAFF) method and combined with pixel-adaptive feature extraction (PAFE) and feature-selective anchor-free detection (FSAD). For food packaging defect detection, Liu et al. [22] designed and integrated the Convolutional Attention Module (CBAM) to enhance attention to key features. They achieved cross-scale feature fusion through pyramid and aggregation networks (enabling capture of defects of different sizes) and added the Adaptive Spatial Feature Fusion module (ASFF) to the backbone network to improve cross-scale feature fusion capability.

**Fig 1:**
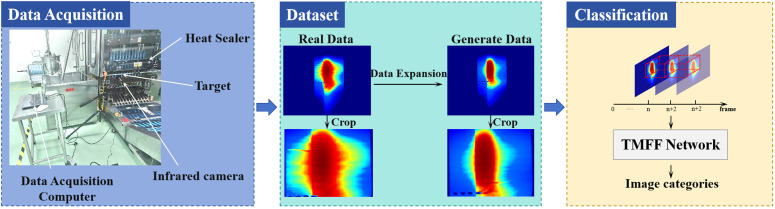
Overall framework of the proposed sealing defect detection system for pharmaceutical plastic bags.

On the basis of the previous work, we propose a comprehensive sealing inspection framework for plastic-sealed bags, which integrates physics-guided data augmentation and a dual-branch temporal network to address class imbalance and improve detection accuracy (Fig 1). This framework first generates physically plausible defective samples by modeling the morphological features and thermal diffusion laws of real defects, effectively mitigating class imbalance without relying on complex generative models. Then, we design a Thermal-Sequence Guided Dual-Branch Network that captures temporal thermal dynamics through global-local feature fusion and channel-aware enhancement, enabling efficient and accurate detection of subtle sealing defects. In summary, our main contributions are as follows:

1. We collected thermal imaging data of heat-sealed pharmaceutical plastic bags and identified that defective sealing regions exhibit distinct thermal characteristics (localized abnormal temperature elevation $>1^{\circ }C$), providing a reliable basis for defect detection.
2. A physics-guided defect synthesis method is proposed to expand defective samples: by modeling defect contours with hybrid polynomials, simulating thermal diffusion via dual-Gaussian operators, and fusing synthetic defects into normal samples under geometric constraints, we achieve high-fidelity data augmentation with low computational cost.
3. A Thermal-Sequence Guided Dual-Branch Network is designed, which incorporates a global-local feature fusion module to enhance sensitivity to small defects and a channel-aware enhancement module (SE-Dense structure) to suppress background noise, enabling robust detection of sealing defects through temporal thermal feature analysis.

## Materials and methods

### Dataset

**Fig 2:**
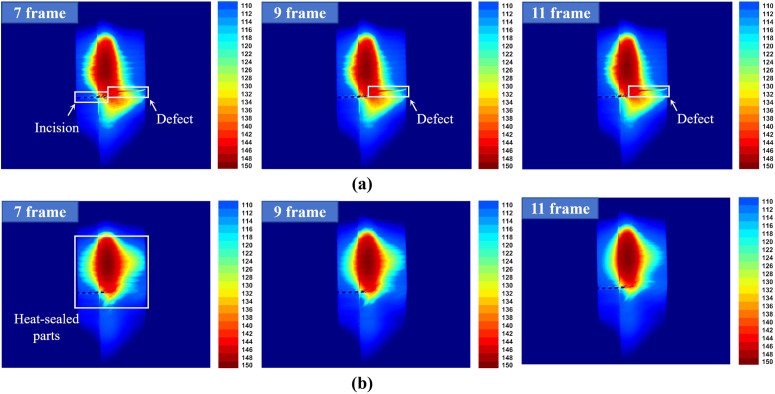
Thermal imaging comparison between normal and defective sealing regions. (a) represents a defective sample, and (b) represents a normal sample.

**Fig 3:**
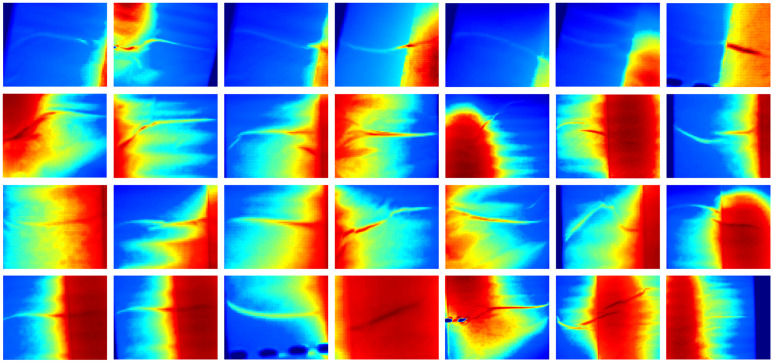
Collection of real sealing defect samples in thermal imaging.

The experimental setup used a Hikmicro HM-TD2C68E-25/Q vanadium oxide uncooled detector (resolution: 640×512, frame rate: 50 Hz, focal length: 25 mm) to capture thermal imaging data during the plastic bag heat-sealing process, the imaging distance was fixed at 10 cm. Data acquisition was performed through video recording, where characteristic frames from each heat-sealing cycle were extracted using a temperature-based feature recognition algorithm. Then manual annotation was performed to determine quality: samples that exhibited a localized abnormal temperature elevation ($>1^{\circ }C$ temperature difference) at the heat-sealed region were labeled leakage defects (Fig 2(a)), while others were designated as normal (Fig 2(b)). The final data set comprises 2,309 heat-sealing cycles, including 2,281 normal samples and only 28 defective samples (Fig 3), demonstrating significant class imbalance.

### Feature-based data expansion

To address the severe class imbalance caused by scarce defective samples in thermal imaging data, this chapter proposes a physics-guided defect synthesis framework that generates thermodynamically plausible samples through three key innovations:

1. An adaptive defect modeling stage where empirical defect contours are extracted via Sobel edge detection and fitted using hybrid polynomial functions (combining both experimentally fitted third-order curves and stochastic fourth-order variations)
2. A thermal diffusion simulation employing dual-Gaussian blurring operators (with parameterized $\sigma_{w}$ and $\sigma_{d}$) to accurately replicate heat dissipation patterns observed in real defects
3. A geometrically constrained fusion process that intelligently integrates the synthesized defect profiles into normal samples. Anatomical correctness is maintained through boundary-aware positioning and manual quality verification, achieving both data expansion and physical fidelity with minimal computational overhead.

### Defect feature modeling

**Fig 4:**
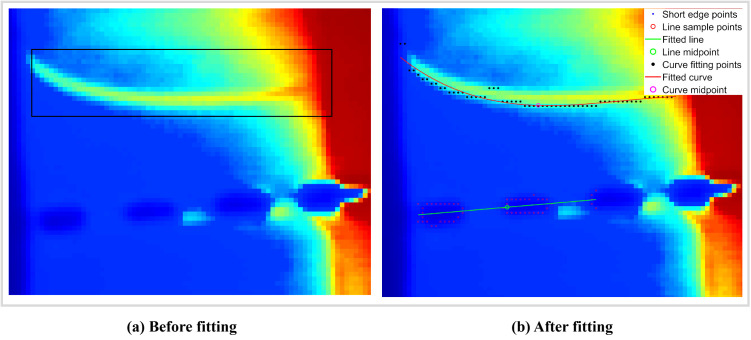
Comparison of defect contour fitting results before and after polynomial fitting.

In the feature extraction stage, the edge of the incision is extracted using the Sobel edge detector. Feature points are then fitted into a line segment, leveraging the incision‘s short length and the characteristic that sampling points form a closed area. After manually selecting the region of interest (ROI), a column scanning process is performed: the pixel with the highest gray intensity in the initial column is designated as the starting feature point of the defect curve. For the following columns, a vertical search window with a fixed height is established, centered on the previous feature point, and the pixel with the largest gray intensity is repeatedly identified as the current feature point in this window (Fig 4).

This local search strategy effectively mitigates deviations induced by image noise and abrupt illumination variations. Upon acquiring sufficient characteristic points, a third-order polynomial is applied to fit the defect curve and derive its analytical expression:


$${ℱ}_{fit}(x)=\sum_{k=0}^{3}a_{k}x^{k}$$ (1)

In addition, the relative spatial coordinates between the midpoint of the incision line segment and the midpoint of the defect-fitted curve are computed, while the coefficients of the defect curve and their spatial relationship to the incision are stored for the construction of the defect feature.

In order to enhance defect feature diversity and simulation robustness, we probabilistically use a random fourth-order polynomial to replace the fitted bright band analytical expression, which is defined as:


$${ℱ}_{\text{rand}}(x)=\sum_{k=0}^{4}b_{k}x^{k}$$ (2)

where coefficients $b_{k}$ follow a scaled normal distribution:


$$b_{k}∼𝒩(0,\sigma^{2})\;\text{with}\;\sigma =50$$ (3)

The final defect curve was generated using a mixed selection strategy. where the defect curve is a randomly selected ${ℱ}_{\text{fit}}^{\left(i\right)}(x)$ from 1 to 4 fitted libraries. Add their coefficients and take the average:


$$\bar{ℱ}(x)=\frac{1}{N}\sum_{i=1}^{N}{ℱ}_{\text{fit}}^{\left(i\right)}(x)\;(N\in \{1,2,3,4\})$$ (4)

The final defect curve 𝒟(x) is probably assigned as:


$$\begin{array}{c} 𝒟(x)=\left\{ \begin{array}{cc} \bar{ℱ}(x) & \text{P}=0.8\\ {ℱ}_{\text{rand}}(x) & \text{P}=0.2 \end{array}\right.\; \end{array}$$ (5)

This hybrid approach preserves empirical fidelity through experimental fits (80% weighting) while incorporating stochastic variations (20% weighting) to improve generalization against unseen defect feature patterns.

### Thermal diffusion simulation

After defect feature modeling, thermal diffusion of the defect model is simulated to replicate the temperature change patterns observed in real defects:

1. **Defect mask initialization:** A binary mask ℳ is initialized to perform the thermal diffusion work, and the pixels on the defect feature curve 𝒞 are assigned as 1.

$$\begin{array}{c} ℳ(u,v)=\left\{ \begin{array}{cc} 1 & \forall (u,v)\in 𝒞\\ 0 & otherwise \end{array}\right.\; \end{array}$$ (6)

where *u* and *v* are the spatial coordinates (unit: pixels) in the image plane

2. **Directional Gaussian blurring:** The spatial propagation of thermal anomalies near defects is modeled via Gaussian-based thermal diffusion blurring of the defect contour mask, with width modulation incorporated. This results in the following expression:

$$𝒲(u,v)=K\cdot \left[\frac{1}{2\pi \sigma_{w}^{2}}\exp\left(-\frac{u^{2}+v^{2}}{2\sigma_{w}^{2}}\right)\right]*\left[ℳ(u,v)\cdot \exp\left(-\frac{u^{2}+v^{2}}{2\sigma_{d}^{2}}\right)\right]$$ (7)

*K* is the mask magnification and is used to adjust the brightness and darkness of the defect curve as a whole, making the generated data closer to the real data. $\sigma_{w}$ is the standard deviation of the Gaussian diffusion nucleus, which controls the range and intensity of thermal diffusion; $\sigma_{d}$ is the standard deviation of the mask modulation nucleus, which controls the attenuation characteristics of the edge of the defect profile.

### Defect data generation

**Fig 5:**
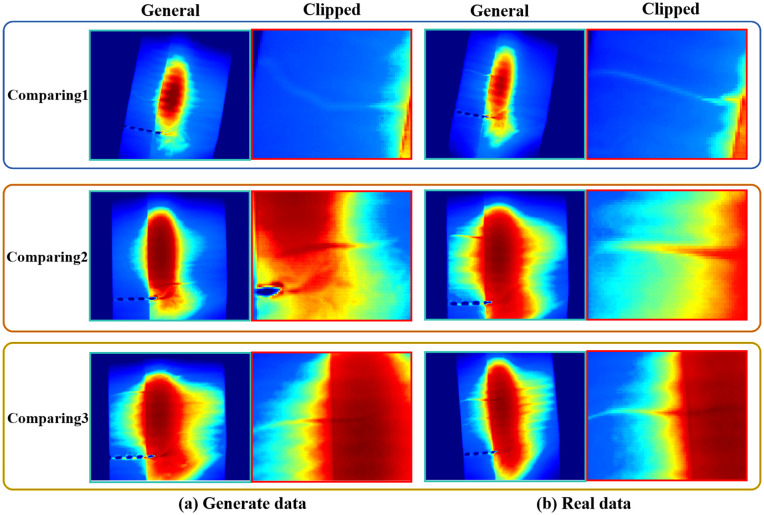
Comparison between real defects and physics-synthesized defects in thermal imaging.

After simulating the thermal diffusion of the defect contours, we obtain a mask where the defect regions closely resemble real defects, while the background remains zero. To prepare the mask for synthesis, we set all zero-value pixels (background) to 1, while keeping the non-zero pixels (defect regions) unchanged. These modified masks are then multiplied with randomly selected normal images (defect-free samples from the dataset) to generate synthetic defect data. The position of each defect is determined on the basis of the stored relative coordinates between the midpoint of the defect curve and the midpoint of the fitted incision line. Since the fitted incision line may slightly deviate from the actual incision position, this introduces a degree of randomness in defect placement, improving the robustness of the synthetic data. However, this offset can sometimes cause the defect curve to extend beyond the edges of the plastic packaging. To address this, we first detect the vertical boundaries of the packaging and then remove any defect curves that fall outside these edges. Finally, we generate the synthetic defect data in batches and manually inspect each sample to ensure quality. The resulting data set is shown in Fig 5.

### Network

Detecting small defects in heat-sealed bags using infrared imaging presents significant challenges because of their minimal spatial extent and similarity to background textures. Conventional CNNs often fail to capture fine defect details due to downsampling, while the subtle differences between defects and wrinkles in infrared images further complicate classification [23]. To address these issues, we propose a dual-branch network based on ResNet [24], with a two-stream input design that simultaneously processes both the full image and a cropped region of interest (ROI). This approach preserves global structural context while forcing the network to focus on local suspicious areas, preventing small defects from being diluted by background noise. An attention mechanism is employed to harmonize global and local feature interactions, avoiding redundancy or conflict [25].

**Fig 6:**
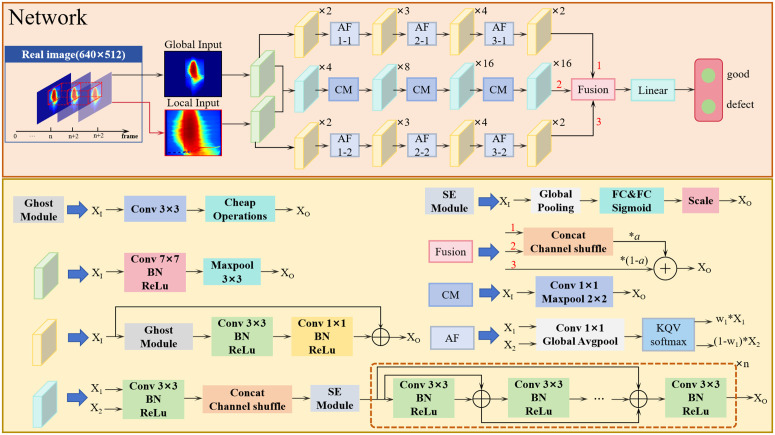
Architecture of the proposed dual-branch temporal network for sealing defect detection.

In addition, because the rapid cooling of heat-sealed bags can lead to changes in the temperature characteristics that mislead classification, we use time-adjacent thermal matrices as input to improve the robustness against thermal drift. The network consists of two complementary branches. The primary branch employs a global-local feature fusion module with an attention mechanism to prevent small defects from being obscured by background information. The secondary branch enhances defect-sensitive channels through a channel-aware enhancement module (SE-Dense structure) to suppress false alarms caused by wrinkles in heat-sealed bags. Following input preprocessing, convolution and pooling operations are applied to the two inputs separately to achieve initial feature encoding. The resulting feature maps are fed into the global-local feature fusion module and channel-aware enhancement module, respectively. Finally, the features from the two modules are weighted and fused to enable classification [26]. The proposed network architecture is illustrated in Fig 6.

### The network architecture

The input structure of our network is as follows. First, three frames of temperature matrices representing the same sample at different time points are obtained. To facilitate the network‘s ability to learn more generalizable features, it is necessary to acquire matrices with distinct temperature variations. Therefore, an interval sampling method is adopted, where sampling is performed every other frame, and the three collected temperature matrices are used as the global input. The global input, initially sized 640×512, is center-cropped to maintain aspect ratio and resized to 256×256. The Local Input is derived by cropping the same region from the three frames of the overall temperature matrices. The cropping region is determined by the midpoint of the detected sealing line segment, which is positioned in the lower left region of the Local Input. Batch processing is enabled through parameterized adjustments. Following input preprocessing, convolution and pooling operations are applied to the two inputs separately to achieve initial feature encoding, laying the foundation for subsequent processing of the temperature matrix feature maps.

### Global-local feature fusion module

Global features alone exhibit limited sensitivity to small defects, necessitating fusion with local features to achieve optimal detection performance. To address this, we introduce a hierarchical attention mechanism that dynamically adjusts the contribution weights of global and local features across multiple scales, enhancing the network‘s sensitivity to subtle anomalies. The core of this module is the Ghost-Residual Block, designed for efficient feature extraction and downsampling. This block integrates a GhostNet-inspired lightweight strategy at both input and output stages [27]: primary convolutions and depthwise separable convolutions collaboratively generate diverse feature maps, reducing parameters by 40% compared to standard convolutions while preserving critical defect patterns.

A residual connection links the block input and output directly. For spatial resolution mismatches, the first Ghost Module handles dimensional alignment, minimizing computation without sacrificing accuracy; for channel mismatches, a 3×3 convolution ensures compatibility. By reducing spatial resolution before expanding channels, the block balances efficiency and feature richness. Its flexibility—adjustable via stride parameters in Ghost Modules and 3×3 convolutions—allows repeated stacking to deepen the network, enabling hierarchical feature extraction. After extraction, global and local features undergo hierarchical attention fusion: feature maps are flattened for secondary processing, and attention weights dynamically assign importance to each feature type, mitigating conflicts, and enhancing sensitivity to small defects. Following four rounds of Ghost-Residual Blocks and attention fusion, two 4×4×512 feature maps are concatenated and subjected to channel shuffling [28], ensuring comprehensive integration and cross-channel information flow.

### Channel-aware enhancement module

The secondary branch focuses on channel-aware enhancement, employing an SE-Dense structure to strengthen defect-sensitive channels and suppress irrelevant background information (e.g., wrinkles), thereby reducing false alarms. This module integrates the SE (Squeeze and Excitation) mechanism with dense connectivity, enabling dynamic highlighting of discriminative features while facilitating rich feature reuse [29,30]. The core of this branch comprises a series of SE-Dense Blocks, each consisting of a channel attention module (SE Module) and a dense feature extraction unit. The SE module first compresses spatial dimensions through adaptive average pooling, then utilizes 1×1 convolutions to learn channel-wise importance weights (with a compression ratio of 4 to balance computational cost and expressiveness). The weights normalized by the hardsigmoid function emphasize channels encoding defect-related patterns while suppressing those dominated by background noise.

As a complement to the attention mechanism, the SE-Dense Block adopts a dense connectivity design: each layer concatenates all preceding feature maps as input, enabling efficient reuse of low-level texture features and high-level semantic cues. The number of channels in the feature maps is doubled via 1×1 convolutions, while the size of the feature maps is halved through pooling operations. This approach retains fine-grained details critical for small defect detection while reducing computational complexity. After preliminary feature extraction via initial convolution and pooling, the features are fed into the SE-Dense Blocks. Following feature extraction, global average pooling compresses spatial information, and a linear layer maps the output to a fixed-dimensional feature space. Through dynamic channel weighting and feature reuse, this branch enhances the network’s ability to distinguish true defects from interfering wrinkles. It complements the global-local fusion of the primary branch, collectively improving the overall robustness of detection.

## Results

Our dataset comprises a total of 4385 samples, including 2281 normal samples and 28 defective samples from the original dataset, along with 2104 generated defective samples. Experimental results demonstrate that our network exhibits excellent generalization capability. In data preprocessing, all images are uniformly resized to 256×256, matching the input size of the model. To validate the generalization performance, two additional industrial defect detection datasets—Glass Bangle Defect Classification [31] and Leather Defect Classification [32]—were incorporated for comparative experiments. We utilized accuracy, precision, recall, and F1-score as evaluation metrics. Specifically, accuracy is defined as the ratio of correctly predicted samples (including both positive and negative classes) to the total number of samples; precision refers to the proportion of correctly predicted samples of a specific class among all samples predicted as that class; recall represents the ratio of correctly predicted samples of a specific class to all actual samples belonging to that class; and the F1-score is a metric that balances precision and recall.


$$\text{Accuracy}=\frac{TP+TN}{TP+TN+FP+FN}$$

$$\text{Precision}=\frac{TP}{TP+FP}$$

$$\text{Recall}=\frac{TP}{TP+FN}$$

$$F1=2\times \frac{\text{Precision}\times \text{Recall}}{\text{Precision}+\text{Recall}}$$

where TP denotes true positive, TN denotes true negative, FP denotes false positive, and FN denotes false negative.

### Network performance comparison

**Fig 7:**
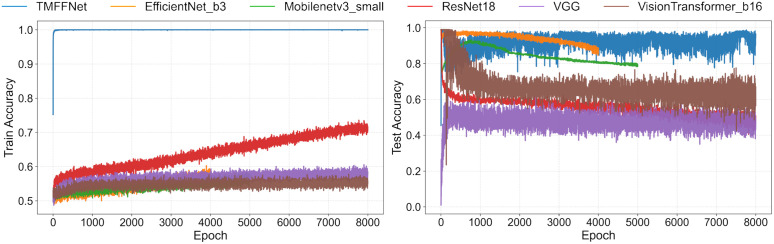
Training and testing of several networks.

We conducted comparative experiments on several classification networks, namely the classic networks VGG [33] and ResNet, the lightweight networks EfficientNet [34] and MobileNet, as well as the self-attention network Vision Transformer. The results demonstrate that the proposed network achieves superior comprehensive performance. Considering the accuracy and recall of negative classes, as well as the overall accuracy of all data, we identify these metrics as key criteria for evaluating network performance. For normal data, the dataset is split in an 8:2 ratio, with 80% of the normal samples used for training and 20% for testing. For defect data, a fixed ratio split introduces significant randomness due to the extreme scarcity of defect samples. Thus, we use all synthetic defect samples for training and retain all real defect samples for testing. The network adopts the cross-entropy loss function, as it is more effective for multi-class classification. The comparative networks are not pre-trained, and their three input channels consist of three frames sampled in one cycle. To ensure valid comparison, the hyperparameters of all networks are set identically. The results of network training and validation are shown in the figure:

The results are shown in Table 1. It can be observed that, compared with other comparative networks, TMFFNet maintains high test accuracy while achieving a high level of recall. Furthermore, during the training process, our network exhibits higher fitting efficiency and shorter computation time. The fitting performance of our network in training is illustrated in Fig 7:

**Table 1:** Performance comparison of different networks.

Network	Accuracy	Precision	Recall	F1-score
TMFFNet	**0.9809**	**0.3077**	0.6667	**0.4211**
VGG16	0.5903	0.0248	**1.0000**	0.0484
ResNet18	0.6493	0.0288	**1.0000**	0.0561
EfficientNet b3	0.8785	0.00294	0.3333	0.0541
MobilenetV3-Small	0.8090	0.0357	0.6667	0.0678
ViT-B/16	0.7292	0.0240	**1.0000**	0.0449

Values represent mean performance. The highest scores in each metric are highlighted in bold.

### Generalization performance verification

Two public datasets covering different defect types were selected, and a comparative analysis of generalization performance was conducted between TMFFNet and three types of representative networks (ResNet18, MobileNetV3 Small, ViT-B/16).

The results are shown in Table 2. The Glass Bangle Defect Detection Classification dataset consists of three classes (good, broken, defect) with 520, 316, and 244 samples respectively. On this dataset, TMFFNet did not achieve the best overall performance; however, it yielded higher Precision and Recall for minority classes, demonstrating better attention to minority samples compared with other networks. For the 5-class Leather Defect Classification dataset, TMFFNet achieved the best performance in all aspects.

**Table 2:** Generalization performance comparison on multiple datasets.

Dataset	Model	Accuracy	Precision	Recall	F1-score
Glass Bangle Defect Detection Classification	TMFFNet	0.8750	0.8736	0.8823	0.8750
	ResNet18	**0.8854**	**0.8843**	**0.8959**	**0.8875**
	MobileNetV3-Small	0.8781	0.8822	0.8646	0.8682
	ViT-B/16	**0.8854**	0.8781	**0.8959**	0.8859
Leather Defect Classification	TMFFNet	**0.9929**	**0.9928**	**0.9927**	**0.9927**
	ResNet18	0.9759	0.9789	0.9752	0.9765
	MobileNetV3-Small	0.9720	0.9883	0.9749	0.9757
	ViT-B/16	0.9616	0.9622	0.9615	0.9616

Each metric represents the average value across all classes. The highest score for each metric is shown in bold.

## Discussion

Our research was conducted under the condition of class imbalance. The proposed TMFFNet outperforms networks such as VGG16 and ResNet18 in terms of comprehensive performance, making it more suitable for practical industrial applications. The extremely high recall rates in some convolutional neural networks, although seemingly impressive, are actually misleading. This perfect recall is likely achieved at the cost of excessive false positives, as evidenced by their very low precision scores. In practical defect detection scenarios, such a high rate of false positives would lead to unnecessary inspections, increased costs, and reduced trust in the system—which makes these networks impractical despite their high recall rates.

In contrast, TMFFNet demonstrates a more balanced performance. It not only achieves the highest test set accuracy but also has a significantly higher precision than the comparative models while maintaining a reasonable recall rate. This balance is crucial for industrial applications, as both accurate identification of defects and minimization of false alarms are equally important. The higher F1-score further confirms TMFFNet’s ability to effectively balance precision and recall. The improved performance of TMFFNet can be attributed to its specialized design. This design better handles the three-frame input structure, enabling the network to capture subtle defect patterns that may be missed by traditional networks. Additionally, the higher fitting efficiency and shorter computation time during training indicate that TMFFNet is not only more accurate but also more practical for deployment in time-sensitive industrial environments. Furthermore, we emphasize the effectiveness of using generated defective samples for training when real defect samples are scarce. By retaining all real defect samples for testing, we ensured a rigorous evaluation of the model’s generalization ability. TMFFNet performed well under this setup, demonstrating its capacity to effectively learn from synthetic data while maintaining good generalization to real-world defects.

However, our research still has several limitations. Due to the use of only three sampled frames within a single cycle as input, the model’s temporal modeling capability remains weak, struggling to effectively capture the temporal characteristics of the data. Additionally, the extremely limited number of real defect samples results in severe class imbalance, which may lead to significant fluctuations in experimental results. Future work will address the temporal modeling limitations of the current three-frame input setup to better capture dynamic temperature changes. We will incorporate more powerful temporal models—such as bidirectional LSTM or Transformer with its inherent temporal attention mechanism—into TMFFNet explicitly. This integration will dynamically weight feature contributions across different time steps, thereby enhancing the model’s capability to detect transient defects with short durations and subtle temperature variations. To tackle the class imbalance problem, we plan to further expand the scale of real defect samples and explore advanced methods more tailored to extremely imbalanced small-sample scenarios in future work, thereby improving the stability and reliability of the model’s predictions.

## Conclusions

In summary, this study addresses the challenges of sealing defect detection in pharmaceutical plastic bags under class imbalance. It proposes an integrated framework combining physics-guided data augmentation and the Temporal Multi-Feature Fusion Network (TMFFNet), and this framework achieves significant advancements in practical industrial applications.

The physics-guided data augmentation method effectively mitigates class imbalance by synthesizing 2104 realistic defective samples based on thermal diffusion laws and defect morphological features. This approach, which avoids complex generative models, ensures high fidelity of synthetic data and lays a solid foundation for model training. Comparative experiments demonstrate that TMFFNet, with its dual-branch design (global-local fusion and channel-aware enhancement), outperforms other networks in comprehensive performance. It achieves a test set accuracy of 0.9809, and other evaluation metrics also show good performance. Additionally, the network exhibits higher fitting efficiency, making it more suitable for time-sensitive production lines.

This framework not only provides a reliable solution for pharmaceutical packaging inspection but also demonstrates potential for extension to other fields such as accessories defect detection and material surface defect detection. However, limitations remain, including room for further improvement in overall performance. Future work will focus on advanced augmentation techniques for more realistic samples, enhancing TMFFNet‘s comprehensive performance, and optimizing its compatibility with high-speed production lines through lightweight designs. Ultimately, this research contributes to advancing non-destructive inspection technologies and improving product safety in manufacturing.
